# Update on pharmacotherapy for ocular surface squamous neoplasia

**DOI:** 10.1186/s40662-019-0150-5

**Published:** 2019-08-12

**Authors:** Ghada Al Bayyat, Dan Arreaza-Kaufman, Nandini Venkateswaran, Anat Galor, Carol L. Karp

**Affiliations:** 1 Bascom Palmer Eye Institute, University of Miami, 900 NW 17th Street, Miami, FL 33136 USA; 2 Miami Veterans Hospital, Miami, FL 33125 USA

**Keywords:** Ocular surface squamous neoplasia (OSSN), Mitomycin-C, Interferon alpha 2b, 5- fluorouracil, Conjunctival neoplasia, Corneal neoplasia

## Abstract

The most frequently encountered non-pigmented tumor of the ocular surface is ocular surface squamous neoplasia (OSSN). Over the past two decades, the pharmacological management of OSSN has grown, with topical 5-fluorouracil, mitomycin, and interferon alpha 2b all being successfully used to treat this disease. Other agents, such as anti-vascular endothelial growth factor (VEGF), retinoic acid, cidofovir and *Aloe vera*, have less frequently been used in the treatment of OSSN. This review will discuss these pharmacologic agents, summarizing available data and presenting the approach to the treatment of these tumors.

## Background

Ocular surface squamous neoplasia (OSSN) encompasses a spectrum of corneal as well as conjunctival lesions ranging in severity from epithelial dysplasia to conjunctival intraepithelial neoplasia in situ to invasive squamous cell carcinoma (SCC). Although OSSN is typically a localized and slow growing tumor, it can rarely invade the sinuses, orbit or the globe [1]. OSSN most often originates from the limbus and lesions are often localized within the interpalpebral fissure, corresponding to areas with sun exposure [2–4]. While the majority of OSSN lesions are non-pigmented, pigmented variants do exist, especially in Africa [5].

### Epidemiology

OSSN incidence has been reported to range from 0.13 to 1.9 per 100,000 individuals, and varies with geography, with higher incidences in areas of greater sun exposure (such as Africa) as compared with areas with less sun (such as Denmark) [6, 7]. Men in their 6th decade of life are commonly affected, but these lesions can also occur in younger individuals, especially when associated with xeroderma pigmentosum (XP) or human immunodeficiency virus (HIV) [8–11]. Studies in Africa report an increased incidence of OSSN in individuals with HIV compared to non-infected individuals [9, 12]. Furthermore, the biology of OSSN is more aggressive in African American individuals with HIV, with more frequent invasion into the orbit than is seen in individuals with HIV in western countries [13].

### Risk factors

Risk factors for OSSN include environmental and genetic factors, with the strongest risk factor being exposure to ultraviolet (UV) radiation. As with cutaneous malignancies, UV can damage DNA and lead to the development of cancer promoting mutations [14]. Individuals with XP are at an increased risk of UV-induced cell damage due to an inability to repair mutations in the DNA [10, 15, 16] and are thus more susceptible to both ocular surface and cutaneous malignancies [17]. Another possible risk factor is the human papilloma virus (HPV) which has been found more frequently in OSSN specimens compared to healthy conjunctiva [18], although the frequency of detection varies with region (low in countries such as India, Germany and Taiwan but high in cities such as Miami, Florida). HPV-induced carcinogenesis has been attributed to the ability of its oncoproteins, specifically E6 and E7, to target and interact with host cellular proteins, such as p53, and to enhance degradation of normal proteins [19]. Finally, immune-suppressive and immune-deviated states, such as in HIV and atopy/allergy, have been associated with OSSN, likely due to impairment in tumor recognition by immune cells [9, 15, 20–22].

## Main text

### Clinical presentation

OSSN typically presents unilaterally, but it can also be bilateral, especially in individuals with XP, HIV, and atopic states/allergy [23]. OSSN has several classic features, which can be present in isolation or in combination, including leukoplakia, opalescence, papillomatous or gelatinous features, abnormal vascularity (presence of hairpin vessels), and irregular corneal borders [10, 24, 25]. OSSN most frequently originates at the limbus and can extend into the cornea or conjunctiva but can involve either of these structures in isolation [24, 25]. Due to the shared risk factor of UV exposure, OSSN can be associated with other ocular surface lesions such as pterygia and pinguecula, and often these lesions can co-exist [26]. Individuals with OSSN can be asymptomatic and their tumors are often identified incidentally on a routine eye examination. Others can present with bothersome symptoms, including persistent tearing, redness, grittiness or pain.

### Diagnostic modalities

**Fig. 1:**
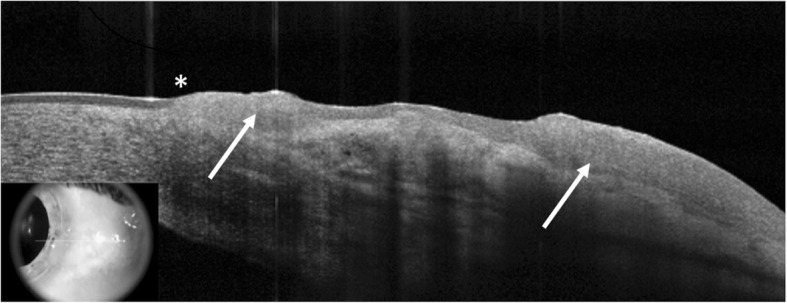
HR-OCT image of OSSN. High-resolution anterior segment optical coherence tomography (HR-OCT) image of a patient with ocular surface squamous neoplasia (OSSN). In this image, the distinct features of OSSN found on HR-OCT can be seen: a thickened, hyperreflective epithelium (white arrows) with an abrupt transition from normal to abnormal epithelium (asterisk)

The diagnosis of OSSN is usually made clinically. Vital dye staining with rose bengal, toluidine blue, or lissamine green can help determine the diagnosis as well as to outline the borders of the tumors [27–29]. Confirmation by pathology after incisional or excisional biopsy is considered the gold standard for diagnosis. Other less invasive modalities including fine needle aspiration biopsy, impression cytology, or exfoliative cytology have been used to sample tissue [24]. Imaging modalities such as high resolution segment optical coherence tomography (HR-OCT), confocal microscopy and fluorescent microscopy have more recently been reported to aid in the diagnosis of OSSN [24, 30–37]. HR-OCT is the imaging modality most frequently used in our institution for the diagnosis and monitoring of OSSN [31–36, 38, 39]. OSSN has a classic appearance on OCT with three characteristic features: (1) hyperreflective, (2) thickened epithelium with (3) an abrupt transition point between normal and abnormal epithelium (Fig. 1). A study of 34 individuals with OSSN or with pterygium found a 94% sensitivity and a 100% specificity of a ultra-high resolution custom OCT device in differentiating OSSN from pterygium, using an epithelial thickness cut off of approximately 140 μm on OCT images [34]. Similarly, another study of 21 lesions of OSSN or of pterygium found a 100% sensitivity and specificity of the RT Vue (Optovue, Fremont, CA) in differentiating these conditions with an epithelial thickness cut off of 120 μm on OCT images [33]. Other studies have shown that distinctive features on HR-OCT have been able to rule-in and rule-out OSSN in the setting of co-existing ocular surface diseases (such as rosacea, limbal stem cell deficiency, mucous membrane pemphigoid, pterygia, pinguecula or Salzmann’s nodular degeneration) that can make the identification of neoplasia challenging [33, 35]. Advantages of OCT are that images are easily and quickly obtained in a non-contact manner. However, limitations of the technology are that the region of scanning is manually determined by the technician and as such, without automated scanning technology of the entire ocular surface, areas of subclinical disease may not be captured. Furthermore, shadowing can obscure the depth of penetration especially in thick tumors and the tumor invasion cannot be detected [24, 25, 32–36]. HR-OCT can also play a role in the ongoing surveillance of OSSN, especially in identifying residual sub-clinical disease despite apparent clinical resolution.

Confocal microscopy has been used less frequently for the diagnosis and monitoring of OSSN, but has shown some utility [30, 37]. On confocal microscopy, large areas of superficial cell and keratin debris can be seen with loss of the normal structure of the conjunctival epithelium and corneal basal epithelium, papillomatous organization and large fibrovascular cores. In vivo cyto-morphologic studies using confocal microscopy can help elucidate abnormalities at the cellular level including high nuclear to cytoplasmic ratios, pleomorphism and hyperreflective and indistinctly bordered cytoplasm [37, 40]. Advantages of confocal microscopy include its ability to examine neoplastic tissue in 5 to 20 μm sections as well as at the cytologic level. Disadvantages include the small area of visualized tissue, the lack of cross-sectional visualization, and the inability to effectively illuminate and reflect through keratinized and necrotic neoplastic tissue. Thus, interpretation of confocal images can be more challenging in tumors than when imaging optically transparent healthy corneal and conjunctival tissue [37, 40].

### Differential diagnosis

Several other lesions must be considered in the differential diagnosis of a corneal or conjunctival OSSN including pterygium, pinguecula, corneal pannus, Salzmann’s nodular degeneration, pyogenic granuloma, as well as amelanotic melanoma [24].

### Treatment

Surgical removal has been considered the gold standard of care for OSSN [41–43]. Surgery has the advantage of serving as both a diagnostic and therapeutic procedure, providing both an accurate histological diagnosis and rapid tumor resolution. However, surgery has potential disadvantages including limbal stem cell deficiency or conjunctival scarring and leaving residual disease that can lead to subsequent tumor recurrence. Given these potential disadvantages, topical chemotherapy has become a popular alternative to surgery [41, 42, 44]. Advantages of topical therapy include treatment of the entire ocular surface which can address areas of subclinical disease, as well as a lower conjunctival scarring risk [45].

### Surgical treatment

Surgical removal is still a widely used approach for the treatment of OSSN, especially in lesions that affect < 4 clock hours of limbus [43]. Excision is generally accomplished with wide margins, typically 4 mm, using a no-touch technique. Cryotherapy of the limbus and conjunctival borders in a double freeze/slow thaw manner and absolute alcohol placement on the limbus are usually performed as adjuvant therapies. If pathological evaluation of the specimen reveals tumor cells at the conjunctival margin, additional surgery or postoperative adjunctive chemotherapeutic drugs may be used after surgery, including interferon alpha 2b (IFNα-2b), 5-fluorouracil (5-FU) 1%, and mitomycin C (MMC) 0.02–0.04%. MMC has also been used as an adjuvant agent intraoperatively [15, 46, 47]. Adjuvant therapies are used both pre- and post-operatively as recurrence rates are higher when margins have residual disease (> 50% in some studies [48]), as compared to when margins are negative for tumor (5 to 33% recurrence frequency with follow-up of 15 years [48]). Table 1 outlines all topical chemotherapy drops and associated side effects.

**Table 1:** Topical chemotherapy for ocular surface squamous neoplasia (OSSN)

Most common OSSN treatment options	Formulation	Dosage	Side effects
IFN-a2b	Topical: 1 MIU/ml (Alternative: 2–3 MIU/ml), Subconjunctival injections: 3 million IU/0.5 ml (Alternative: 10 MIU/month)	Topical: 4 times a day drops continuously, Subconjunctival: Weekly injections until resolution (typically 4–5 weeks)	Minimal side effects for drops, flu-like malaise with injections
5-FU	Topical: 1% drops	4 times a day for 1 week with 3 weeks off (Alternative: 4 times daily for 2 days to 4 weeks)	Mild pain, lid edema, epitheliopathy
MMC	Topical: 0.02–0.04% drops	4 times a day for 1 week followed by 2–3 weeks off until the eye is quiet. Usually 3–4 cycles until resolution (Alternative: 7–14 day cycles)	Pain, keratopathy, punctal stenosis, LSCD

*LSCD*= limbal stem cell deficiency; *MIU*= million international units; *LSCD*= limbal stem cell deficiency; *MIU*= million international units; *IFN-a2b= * interferon alpha 2b; * 5-FU=* 5-fluorouracil; *MMC= * mitomycin C

### Interferon alpha 2b (IFNα-2b)

#### Pharmacology

Interferons are leukocyte-derived proteins that can enhance phagocytic and cytotoxic mechanisms, inhibit biosynthetic enzymes, decrease blood vessel proliferation, induce apoptosis and inactivate viral RNA. Interferon α-2b (IFNα-2b) specifically is a cytokine containing 165 amino acid residues with immunomodulatory effects. Intralesional injections of IFNα-2b enhance the production of IL-2 and IFN-γ mRNA by the immune system and lower the production of IL-10. These mechanisms help in the recognition and targeting of neoplastic cells [49].

#### Treatment of OSSN

**Fig. 2:**
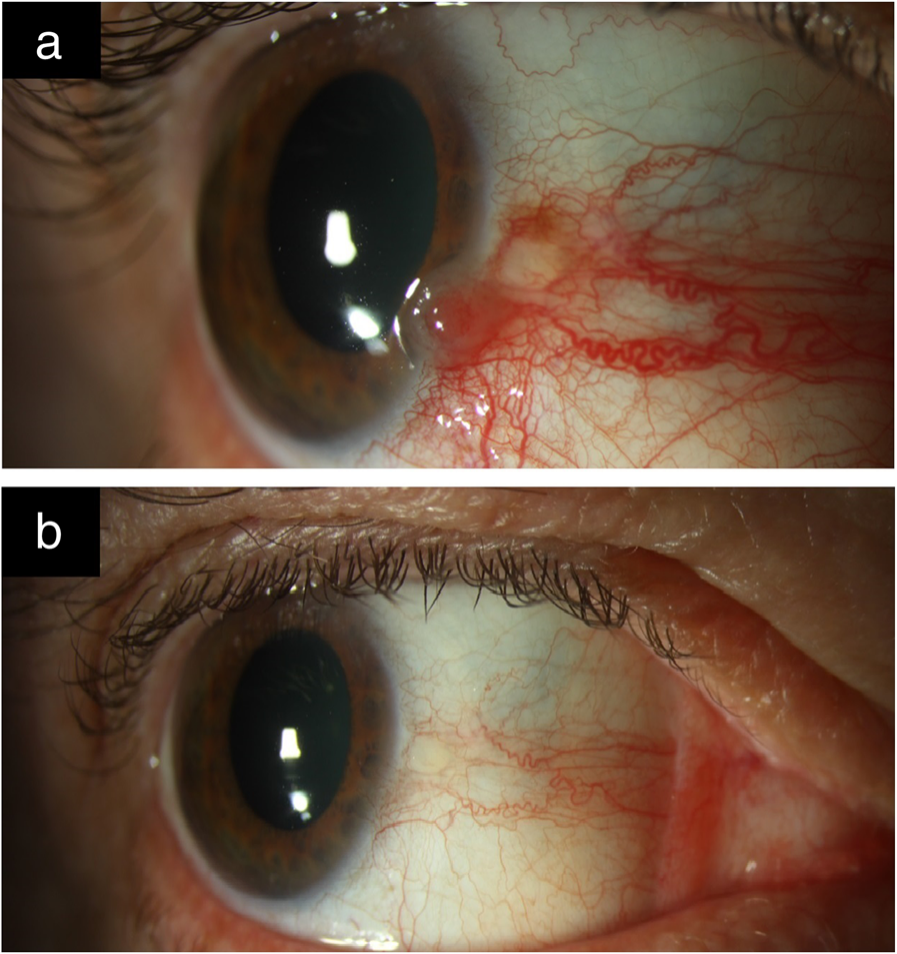
Slit-lamp photo of OSSN before and after IFNα-2b treatment. **a**. Slit-lamp picture of the right eye with ocular surface squamous neoplasia (OSSN). There is an elevated limbal gelatinous mass with extension of the tumor onto the corneal surface. **b**. Slit-lamp picture of the same patient after treatment with topical interferon alpha 2b (IFNα-2b) 1 million IU/ml four times daily for 3 months. There is complete resolution of the conjunctival/cornea neoplasia. Treatment was used for four months in total

When used to treat OSSN, IFNα-2b for OSSN can be used as topical eye drops, subconjunctival injections, or a combination of both [50–54]. Topical and intralesional IFNα-2b can be used as primary or adjuvant therapies (Fig. 2a and b).

#### Dosage

Topically, the most frequently prescribed IFNα-2b dose is 1 million IU/ml. Typically, the medication is used four times a day without interruption until one or two more months after clinical resolution of the lesion. The average time for clinical resolution is about 4 months [10, 25, 50, 53]. Other doses, including 2 and 3 million IU/ml, have also been reported and are used in the same manner. In one study, a similar efficacy was found between the 1 million IU/ml and 3 million IU/ml doses, with no statistically significant differences in clinical resolution and duration of treatment [53]. When used as a post-surgical adjuvant in individuals with positive margins, topical IFNα-2b 1 million IU/ml is administered four times daily for a duration of 2 months post-operatively [3]. Subconjunctival IFNα-2b injections (3 million IU/0.5 ml) are administered weekly until OSSN resolution (typically requiring 4 to 5 injections to achieve clinical resolution) [50, 52, 54]. Higher concentrations have also been reported, the highest being 10 million IU given monthly [25, 54]. Topical eye drops have the advantage of ease of use and a minimal side effect profile. Subconjunctival injections have more side effects than the drops (e.g. flu-like symptoms) but have the benefits of decreased out-of-pocket costs (because the medication is usually covered by insurers), quicker resolution, no need for compounding, and assured compliance [50].

#### Side effects

When administered topically, IFNα-2b eye drops generally have minimal to no side effects. However, hyperemia and follicular conjunctivitis have infrequently been reported along with formation of epithelial microcysts [55]. The main side-effect associated with subconjunctival injections is a flu-like syndrome that lasts for approximately 48 h after administration of the injection. To lessen the severity of the side effects, 1 g of oral acetaminophen is administered at the time of the injection and then as needed until symptoms resolve [10, 25, 50, 52, 56].

#### Efficacy and cost

Many studies have looked at the use of topical interferon as primary treatment for OSSN. In a study of 24 eyes using topical IFNα-2b (1 million IU/mL) 4 times daily, 22 patients (92%) had complete resolution with treatment and 2 patients (8%) (2/22) did not respond to treatment. The mean time to resolution was 3.25 months and the group was followed for a mean of 18 months [57]. In another case series of 22 patients (17 on IFNα-2b eye drops (1 million IU/mL), 1 receiving subconjunctival injections (10 million IU/ml), and 4 on a combination of therapy, 18 (82%) of tumors resolved completely with treatment and 4 (18%) showed a partial response [54]. In a study of 98 eyes, topical IFNα-2b (*n* = 49) was compared to surgical excision (*n* = 49). Of the 49 eyes receiving chemotherapy, 40 eyes were treated with topical IFNα-2b (1 million IU/ml or 3 million IU/ml), 1 received subconjunctival injections (3 million IU in 0.5 ml), and 8 eyes received both modalities. Recurrence rates were similar between the groups with a 1 year OSSN recurrence rate of 3% in the IFNα-2b group and 5% in the surgery group. In both treatment groups, side effects were mild [58]. Downsides of topical IFNα-2b include the need for refrigeration and the high costs to compound (approximately $600 for a month supply in the USA) [25]. In other countries, IFNα-2b may be quite inexpensive. In the USA, these compounded medications are generally out of pocket costs for the patient. Subconjunctival injections are usually covered by insurers and they are available commercially without the need for compounding.

#### Alternative uses

IFNα-2b has been used to treat other types of tumors including cervical intraepithelial neoplasia, actinic keratosis and metastatic malignant melanoma [59, 60]. Interferon has also been used to treat infections such as chronic hepatitis B and C [61, 62]. In the field of ophthalmology, interferons have also been used to treat conjunctival papillomas, lymphoma, and conjunctival Kaposi sarcoma [63–67].

### 5- fluorouracil (5-FU)

#### Pharmacology

5-FU is a pyrimidine analog that blocks thymidine synthase, which inhibits DNA formation. This leads to a reduction in RNA, which causes poor cell growth and cell death [68].

#### Treatment of OSSN

**Fig. 3:**
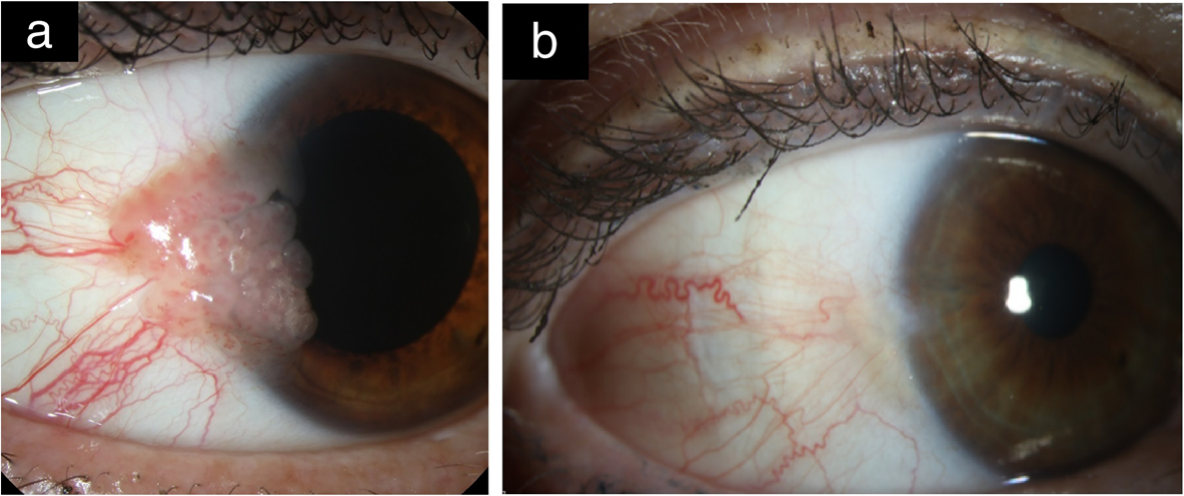
Slit-lamp photo of OSSN before and after 5-FU treatment. **a**. Slit-lamp picture of right eye with a large papillary OSSN located temporally abutting the limbus with extension onto the cornea. **b**. Slit-lamp picture after 4 cycles of 5-fluorouracil (5-FU) 1% shows the clinical regression of the papillary OSSN. There is a small underlying remaining pterygium

5-FU is a well-tolerated and effective agent in the primary treatment of OSSN as well as an adjuvant after surgical excision [69–71] (Fig. 3a and b).

#### Dosage

5-FU is compounded as a 1% solution and is typically used in a cyclical pattern four times daily for a week followed by a 3 week holiday [69, 71]. This pattern makes one cycle, which is repeated on average of 4 to 6 cycles based on clinical response (noted by both slit lamp examination and HR-OCT imaging). Alternative regimens have been reported in the literature, including continuous administration of 5-FU 3 to 4 times a day for 4 weeks or administration of 5-FU for only 2 to 4 days with a 30 to 45 day drug-holiday. [45, 47, 70, 72–74]. 5-FU is preferably kept in refrigeration [69].

#### Side effects

When comparing side effects of topical 5-FU with IFNα-2b, 5-FU has more side effects, most frequently pain and redness at the instillation side, but 5-FU has fewer side effects than MMC (discussed below). Other side effects of 5FU include eyelid swelling, conjunctival congestion, filamentary keratitis and rarely superficial stromal melting [25, 69, 71, 72]. To reduce symptoms, topical corticosteroids are used in conjunction with preservative free artificial tears. Punctal or canalicular stenosis can occur with systemic 5-FU treatment but not with topical 5-FU use [25].

#### Efficacy and cost

In a study of 44 eyes, 5-FU 1% was used as a primary therapy for the treatment of OSSN, administered 4 times daily for a week followed by 3 weeks of no treatment for a mean of 4 cycles [69]. With this therapy, 36 eyes (82%) had complete tumor resolution, 4 (9%) had partial resolution, and 4 (9%) had no response. In those with a complete resolution, the recurrence rate was 11% (4/36) at 2 years with a mean follow up time of 10 months [69]. In another study of 22 eyes, 5-FU 1% was used four times a day continuously for 4 weeks as primary therapy of OSSN [70] and found that all eyes had complete tumor resolution. However, 3 eyes (14%) experienced a recurrence and were re-treated with additional doses of chemotherapy over a follow up time of 63 to 122 months [70]. A large study compared topical 5-FU 1% to topical IFNα-2b for the primary treatment of OSSN in over 100 patients [71] and found comparable results between the two drugs. 52 of 54 eyes (96%) had complete resolution with 5-FU 1% compared to 39 of 48 eyes (81%) with IFNα-2b [71]. A 1 month treatment of 5-FU costs approximately $38 in the US [25, 71].

#### Alternative uses

5-FU can be used as treatment for colorectal cancer, actinic keratosis, as well as head and neck solid tumors [75–77]. 5-FU also has others uses in the field of ophthalmology, namely as an adjuvant agent in glaucoma, pterygium, and vitreoretinal surgery. It is also is used in the post-operative period to revive failing glaucoma blebs [78].

### Mitomycin C (MMC)

#### Pharmacology

Mitomycin is an alkylating agent with antineoplastic/antibiotic properties. It binds to DNA during DNA synthesis and causes inhibition of its synthesis and function [79]. At higher concentrations, MMC inhibits nucleotide synthesis which interferes with RNA transcription and protein synthesis. MMC is toxic to proliferating and non-proliferating cells by inducing apoptosis and inhibition of fibroblast migration [80, 81].

#### Treatment of OSSN

**Fig. 4:**
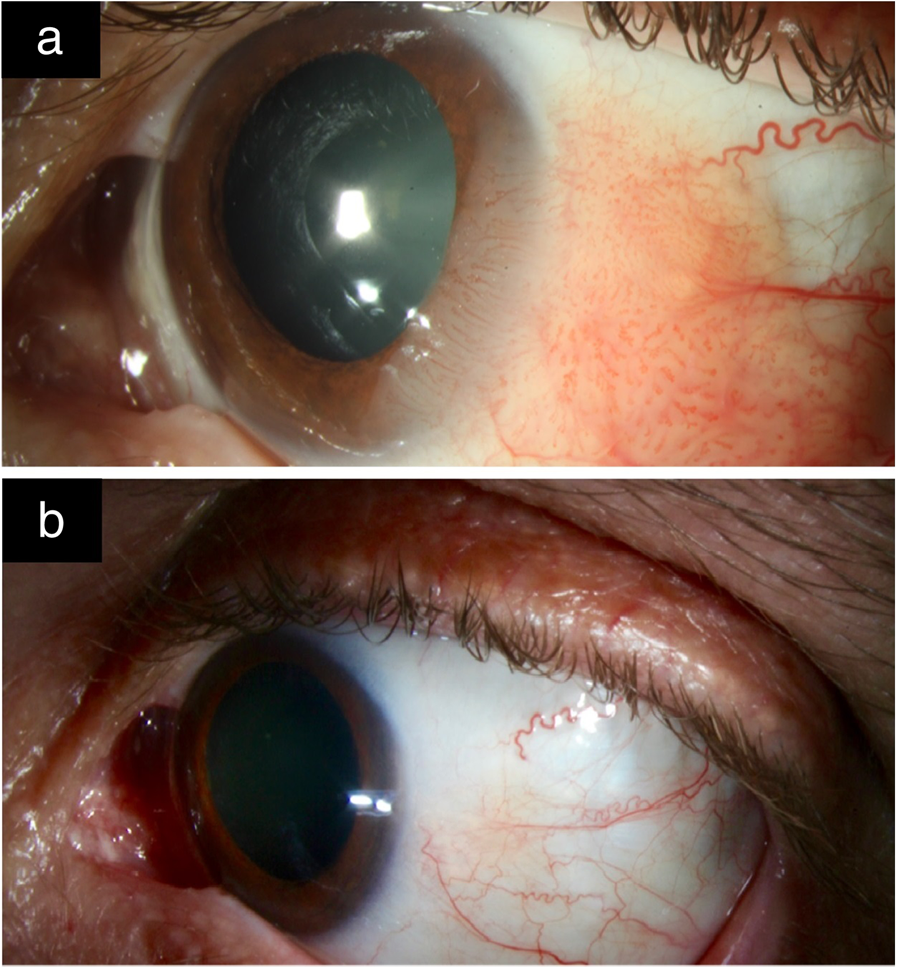
Slit lamp photo of OSSN before and after MMC treatment. **a**. Slit-lamp picture left eye with an extensive OSSN. Papillary fronds of this diffuse OSSN are noted on the bulbar, limbal and corneal surface. **b**. Slit-lamp picture after 3 weekly cycles of mitomycin-C (MMC) 0.04% with 2 to 3 weeks between cycles. The OSSN notably regressed with treatment

MMC can be used as a primary treatment agent for OSSN [82–85] but can also be used intraoperatively, as an adjuvant to excisional biopsy, and post-operatively in patients with positive conjunctival or deep margins [82, 86] (Fig. 4a and b).

#### Dosage

When used as a primary therapy for OSSN, MMC is generally compounded into a concentration between 0.02 and 0.04% [25, 82–85, 87] although one group used a lower concentration (0.002%) [88]. MMC is used 4 times daily for 1 week followed by 2 to 3 weeks of no treatment. The length of the holiday depends on how long it takes the eye to recover from the 1 week of treatment. Other regimens include 1 week on/1 week off, 2 weeks on with variable duration between cycles [10, 83, 89], and 3 weeks of continuous therapy [87]. It is not known which of these concentrations and/or regimens is optimal in maximizing efficacy while minimizing toxicity [85]. MMC can also be used intraoperatively during tumor excision, typically soaked on a sponge at a concentration of 0.02% and applied to the conjunctival margins for a duration of 1~3 min [84, 90, 91]. Postoperatively, in patients with positive margins, MMC drops can be used after the surface heals. The dose and protocol during post-operative use is similar to that of primary use [92]. MMC refrigerated at a temperature of (4 °C) [93].

#### Side effects

MMC has more frequent and severe adverse effects when compared to IFNα-2b or 5-FU, including frequent pain and epitheliopathy. In addition, allergic conjunctivitis, hyperemia, ectropion [10, 94], and punctal stenosis have all been described [95]. Due to the risk of punctal stenosis, punctal plugs should be inserted prior to initiating topical MMC [95]. The 2 to 3 week medication holiday in each cycle helps with patient comfort and compliance.

#### Efficacy and cost

A large randomized controlled trial of 48 patients with OSSN compared MMC use as primary therapy to placebo. MMC 0.04% (0.4 mg/ml) was administered four times a day for 3 weeks. In the 26 patients treated with MMC, 24 (92%) had complete clinical resolution and 2 (8%) had no response. In the placebo group, none of the 22 individuals responded to therapy over the 8 week study period [87]. In 18 eyes, MMC 0.02% was used four times a day continuously for 28 days as primary treatment for OSSN with complete resolution in all patients. One recurrence was noted after 24 months [89]. In 20 eyes with OSSN (3 primary lesions and 17 recurrent lesions) treated with MMC 0.02% (*n* = 6) or 0.04% (*n* = 14) four times a day for an average of 2 cycles (1 week on/1 week off), the mean time to resolution was 4.5 weeks in the 18 (90%) eyes with complete resolution. Overall, four recurrences were noted over an average follow-up period of 57 weeks, 2 of which were successfully retreated with MMC and 2 with alternative therapies [96]. In 7 eyes, MMC 0.04% was used for primary OSSN, given four times a day in cycles of 1 week on/1 week of until tumor resolution (average 2 cycles) [97]. Six eyes (85%) had complete resolution and 1 partial resolution with a mean follow up time of 9 months [97].

In a study comparing MMC 0.04 mg/ml to IFN 1 million IU/ml, similar rates of clinical resolution were found (92% with MMC with 89% with IFN); however, statistically significant shorter resolution times were noted with MMC (median 1.5 months) as compared with IFN (mean 3.5 months), *p* < 0.0005. Unfortunately, patients treated with MMC (88%) experienced significantly more adverse effects compared to those patients treated with IFN (12%), *p* < 0.0005 [98].

Similarly, in a study that used either 5-FU 1% or MMC 0.04% for the primary treatment of diffuse OSSN, both 5-FU and MMC caused drug-related complications in approximately 58% of treated cases (7 of 12 cases with 5-FU and 23 of 39 cases with MMC). This study also showed that diffuse disease (involving > 5 limbal clock hours or extensive corneal spread) was recalcitrant to only one treatment cycle of either 5-FU or MMC and required additional treatment cycles [81]. As seen from these studies, MMC while effective has an unfavorable side effect profile; as such, authors of this paper preferto use IFN or 5-FU as initial treatment modalities, whenever possible. MMC is often reserved for more recalcitrant cases that have failed prior therapy with alternative agents. MMC costs about $100–190/cycle in the United States but is inexpensive outside of the US.

#### Alternative uses

MMC can be used for the treatment of squamous cell carcinoma of the bladder, gastric, pancreatic and colorectal carcinoma [99–102]. MMC has other uses in the field of ophthalmology including as an adjuvant agent in pterygium, glaucoma, refractive, and oculoplastic (dacryocystorhinostomy) procedures [80].

### Retinoic acid

#### Pharmacology

Vitamin A (retinol), and retinoic acid are involved in various cellular processes such as differentiation and growth. Retinoids have potent antineoplastic effects, including regulation of cellular growth by blocking cell maturation and modulating apoptosis [103].

#### Treatment of OSSN

In limited studies, retinoic acid has been used alone or in conjunction with IFNα-2b for OSSN treatment [104, 105]. Given that most studies used it in combination with IFNα-2b, it is difficult to elucidate the independent effect of retinoic acid on OSSN [104–107].

#### Dosage

In one case report, topical retinoic acid 0.01% was applied once every other day for 9 months [107]. In another case, topical retinoic acid 0.01% was applied once every other day in conjunction with topical IFNα-2b until clinical resolution and then continued for a duration of up to 12 months [105]. Another study reported on retinoic acid ointment 0.05% used 1–3 times a day for 1 to 2 weeks [104].

#### Side effects

Retinoic acid has been reported to cause eye lid irritation [104], epithelial microcysts and marginal keratitis [105]; however, side effects are overall mild.

#### Efficacy and cost

In the largest series to date, retinoic acid 0.01% was used in 89 patients once every other day in combination with topical IFNα-2b 1 million IU/ml drops 4 times daily. Complete clinical resolution was noted in 98% (87/89) and partial response was noted in 2% (2/89). Four tumor recurrences were noted over a mean follow up of 4 years [105]. Another study reported two patients who used retinoic acid ointment 0.05%, applied 3 times a day for 10 weeks. Both eyes experienced complete tumor response within 4 weeks [104]. Each 5 ml of 0.01% retinoic acid costs approximately $75 USD.

#### Alternative uses

Retinoic acid has been used in the dermatologic field both topically and systemically to treat moderate to severe acne and psoriasis [108]. It can be used systemically in acute promyelocytic leukemia [109]. In the eye, it has been used to treat ocular consequences of vitamin A deficiency (e.g. keratomalacia) [110].

### *Aloe Vera*

#### Pharmacology

Aloe plants demonstrate antiproliferative, immunostimulatory and antioxidant properties. Aloe consists of the molecules aloin and aloe emodin which mediate the anti-proliferative activity [111, 112]. Studies have shown that aloe emodin can induce apoptosis in vitro [112]. Acemannan is a D-isomer mucopolysaccharide in *Aloe vera* with immunostimulatory effects, likely inducing an anti-neoplastic outcome [111, 112] .

#### Treatment of OSSN

There has been one report on the use of skin *Aloe vera* drop as a primary treatment modality of OSSN [112].

#### Dosage

*Aloe vera* oil drops were used three times daily for 3 months [112]. In this case report, a commercially available formulation of *Aloe vera* was used: Aloe Fleur de Jouvence (Forever Living Products; Scottsdale, AZ).

#### Side effects

No side effects were reported with the topical use of *Aloe vera* [112].

#### Efficacy and cost

In this case, *Aloe vera* drops used three times daily led to a complete clinical resolution after 3 months. No recurrence was noted for 6 years [112]. One bottle of *Aloe vera* oil costs approximately $25.

#### Alternative uses

*Aloe vera* has been used orally and topically to treat different dermatological skin conditions (e.g., burns, frostbite, psoriasis, sunburn), osteoarthritis and other inflammatory processes [113].

### Cidofovir

#### Pharmacology

Cidofovir is a monophosphate nucleotide analogue that demonstrates in vitro activity against a number of DNA viruses [114]. Its clinical activity has been widely demonstrated against cytomegalovirus infection. Since HPV has been implicated in the etiology of OSSN, the anti-viral effects of cidofovir may underlie the efficacy seen in the one published case in OSSN [115].

#### Treatment of OSSN

One case report demonstrated the use of topical cidofovir as primary treatment for OSSN [115].

#### Dosage

Topical cidofovir eye drops (2.5 mg/ml) were administered six times a day for 2 weeks, then four times daily for 2 weeks, and then three times daily for 2 weeks to treat diffuse superior tarsal OSSN. The majority of the lesion resolved and the remaining focal area of disease at the nasal edge of the tarsus was surgically excised with adjuvant cryotherapy. No recurrences were noted after 2 years of follow up [115].

#### Side effects

In this one case, cicatrization of the inferior punctum was reported [115]. Ocular surface irritation, conjunctival erythema, and pain are the main side effects of topical cidofovir, which typically resolve after drop cessation.

#### Efficacy and cost

Cidofovir is a compounded medication and costs approximately $300–400 USD for one vial (75 mg/ml, 5 ml) in the US.

#### Alternative uses

Cidofovir has been used as a treatment for various DNA viruses. Topical cidofovir is used to treat herpes viruses, pox viruses, and papilloma viruses of the skin that do not respond to conventional therapies [116]. Cidofovir has also been applied to molluscum contagiosum and condylomata acuminata lesions. Oral cidofovir is used to treat mucosal neoplasms thought to have a viral etiology, such as squamous papilloma of the pharynx and esophagus and Kaposi’s sarcoma [117]. In the eye, cidofovir has been used to treat CMV retinitis through intravitreal injections [117, 118].

### Anti-vascular endothelial growth factor (VEGF)

#### Pharmacology

Anti-VEGF agents are monoclonal antibodies that block the interaction of vascular endothelial growth factor (VEGF) and its receptor, interfering with the growth of blood vessels [119]. Bevacizumab is a complete anti-VEGF antibody while ranibizumab is a fragment of the antibody [119].

#### Treatment of OSSN

There have been a few case reports on the use of anti-VEGF agents as primary therapy in OSSN and as adjuvants after surgical excision [120–123]. Therefore, their role in treating OSSN remains uncertain.

#### Dosage

Dosages of anti-VEGF therapy vary between studies. As primary therapy in OSSN, topical bevacizumab (5 mg/ml) was given 4 times a day for up to 8 weeks [120] and subconjunctival/perilesional bevacizumab (2.5 mg in 0.1 ml) was given weekly for 2 weeks [121]. Lower concentrations of subconjunctival bevacizumab (1.25 mg in 0.05 ml) have also been reported [122]. In another study, subconjunctival ranibizumab (0.05 mg of 10 mg/ml) was injected once or twice a month for a period ranging from 6 to 25 months [123].

#### Side effects

No side effects have been reported with the use of anti-VEGF agents in OSSN. However, bevacizumab may have inhibitory effects on corneal healing, especially when used in patients with epithelial defects [120, 124].

#### Efficacy and cost

A few studies have looked at the role of anti-VEGF agents in the primary treatment of OSSN and as an adjuvant to surgery. In one study, ten patients with primary OSSN diagnosed by impression cytology received 2 perilesional/subconjunctival injections of bevacizumab (2.5 mg/0.1 ml), one week apart [121]. Of these ten patients, two eyes with conjunctival OSSN had complete tumor resolution while eight eyes with corneal OSSN had no significant change to the lesion. In another study of six eyes, topical bevacizumab (5 mg/ml) was given 4 times daily as eye drops for a period of 8 weeks [120]. Two patients (34%) had complete OSSN resolution while 4 (66%) had partial resolution and underwent excisional biopsy. No recurrences were noted after 6 months of follow up [120]. Another case series of five patients were treated with perilesional ranibizumab (0.05 mg of 10 mg/ml) once or twice a month for an average of 19 months (ranging from 6 to 25 months) [123]. Three (60%) had complete resolution and 2 (40%) had partial tumor resolution [123]. Intralesional bevacizumab (1.25 mg/0.05 ml) was also used in a patient with OSSN that failed treatment with MMC, 5-FU and IFNα-2b eye drops [122]. No change in OSSN was noted after the intralesional injection of bevacizumab [122].

#### Alternative uses

Bevacizumab is approved by the FDA for the treatment of colorectal cancer [125]. In ophthalmology, it is used off-label in age-related choroidal neovascularization, proliferative and non-proliferative diabetic retinopathy, neovascular glaucoma, and anterior segment neovascularization [126–128]. Similarly, ranibizumab is FDA approved for the treatment of patients with wet age-related macular degeneration, macular edema following retinal vein occlusion, diabetic macular edema, diabetic retinopathy, and myopic choroidal neovascularization.

## Conclusion

In summary, great advances in the management of OSSN have occurred with the development of numerous topical therapies, and the ability to diagnose and follow the disease with HR-OCT. Fortunately, OSSN is a neoplastic disorder amenable to treatment with topical chemotherapeutic agents. The most commonly used agents in OSSN are IFNα-2b, 5-FU, and MMC, with robust evidence in the literature on their efficacy; less information is available on other agents such as cidofovir, anti-VEGF and retinoic acid. In the authors’ clinical practice, 5-FU is used as first line therapy due to great efficacy, mild side effects and affordable costs. In the rare occasions that 5-FU does not work and causes side effects due to a fragile ocular surface, IFNα-2b is a great alternative due to a similar efficacy profile and milder side effects. Its use is often limited by cost and access to the drug. Interestingly, IFNα-2b may have decreased efficacy compared with 5-FU in patients with underlying immunosuppression [129]. In our center, MMC is considered a second line agent for OSSN when the others fail because of its unfavorable side effect profile, notably corneal toxicity, limbal stem cell deficiency, and punctal stenosis. Surgery is still an excellent option in patients with small lesions (< 4 clock hours) or with rapidly growing lesions and allows for rapid resolution of disease. Topical chemotherapy however, is preferred in large and multifocal lesions, allowing for complete treatment of the ocular surface and a decreased risk for subsequent limbal stem cell deficiency. With topical treatment, the duration is longer and requires compliance. Overall, eye care providers need to consider patient preferences, cost, and access to care when deciding on a treatment approach; however, the pendulum is swinging in favor of the use of topical drugs in the management of ocular surface squamous neoplasia.

## References

[1] Santoni A, Thariat J, Maschi C, Herault J, Baillif S, Lassalle S (2019). Management of invasive squamous cell carcinomas of the conjunctiva. Am J Ophthalmol.

[2] Murillo JC, Galor A, Wu MC, Kye NK, Wong J, Ahmed IO (2017). Intracorneal and intraocular invasion of ocular surface squamous neoplasia after intraocular surgery: report of two cases and review of the literature. Ocul Oncol Pathol..

[3] Kao AA, Galor A, Karp CL, Abdelaziz A, Feuer WJ, Dubovy SR (2012). Clinicopathologic correlation of ocular surface squamous neoplasms at Bascom palmer eye institute: 2001 to 2010. Ophthalmology..

[4] Pe'er J. Ocular surface squamous neoplasia. Ophthalmol Clin North Am. 2005;18(1):1–13, vii. 10.1016/j.ohc.2004.08.001 15763187

[5] Shields CL, Manchandia A, Subbiah R, Eagle RC, Shields JA (2008). Pigmented squamous cell carcinoma in situ of the conjunctiva in 5 cases. Ophthalmology..

[6] Lee GA, Hirst LW (1995). Ocular surface squamous neoplasia. Surv Ophthalmol.

[7] Shields CL, Chien JL, Surakiatchanukul T, Sioufi K, Lally SE, Shields JA (2017). Conjunctival tumors: review of clinical features, risks, biomarkers, and outcomes--The 2017 J. Donald M. Gass Lecture. Asia Pac J Ophthalmol (Phila).

[8] Ramberg I, Heegaard S, Prause JU, Sjö NC, Toft PB (2015). Squamous cell dysplasia and carcinoma of the conjunctiva. A nationwide, retrospective, epidemiological study of Danish patients. Acta Ophthalmol.

[9] Gichuhi S, Sagoo MS, Weiss HA, Burton MJ (2013). Epidemiology of ocular surface squamous neoplasia in Africa. Tropical Med Int Health.

[10] Tsatsos M, Karp CL (2014). Modern management of ocular surface squamous neoplasia. Expert Review of Ophthalmology.

[11] Carreira H, Coutinho F, Carrilho C, Lunet N (2013). HIV and HPV infections and ocular surface squamous neoplasia: systematic review and meta-analysis. Br J Cancer.

[12] Rathi SG, Ganguly Kapoor A, Kaliki S (2018). Ocular surface squamous neoplasia in HIV-infected patients: current perspectives. HIV AIDS (Auckl).

[13] Gichuhi S, Ohnuma S, Sagoo MS, Burton MJ (2014). Pathophysiology of ocular surface squamous neoplasia. Exp Eye Res.

[14] McClellan AJ, McClellan AL, Pezon CF, Karp CL, Feuer W, Galor A (2013). Epidemiology of ocular surface squamous neoplasia in a veterans affairs population. Cornea..

[15] Kiire CA, Srinivasan S, Karp CL (2010). Ocular surface squamous neoplasia. Int Ophthalmol Clin.

[16] Gupta N, Sachdev R, Tandon R (2011). Ocular surface squamous neoplasia in xeroderma pigmentosum: clinical spectrum and outcome. Graefes Arch Clin Exp Ophthalmol.

[17] Suarez MJ, Rivera-Michlig R, Dubovy S, Rodriguez FJ (2015). Clinicopathological features of ophthalmic neoplasms arising in the setting of xeroderma pigmentosum. Ocul Oncol Pathol.

[18] Scott IU, Karp CL, Nuovo GJ (2002). Human papillomavirus 16 and 18 expression in conjunctival intraepithelial neoplasia. Ophthalmology..

[19] Mammas IN, Sourvinos G, Giannoudis A, Spandidos DA (2008). Human papilloma virus (HPV) and host cellular interactions. Pathol Oncol Res.

[20] Karp CL, Scott IU, Chang TS, Pflugfelder SC (1996). Conjunctival intraepithelial neoplasia. A possible marker for human immunodeficiency virus infection?. Arch Ophthalmol.

[21] Ferenczy A, Coutlée F, Franco E, Hankins C (2003). Human papillomavirus and HIV coinfection and the risk of neoplasias of the lower genital tract: a review of recent developments. CMAJ..

[22] Wang CJ, Sparano J, Palefsky JM (2017). Human immunodeficiency virus/AIDS, human papillomavirus, and anal cancer. Surg Oncol Clin N Am.

[23] Gichuhi S, Macharia E, Kabiru J, Zindamoyen AM, Rono H, Ollando E (2016). Risk factors for ocular surface squamous neoplasia in Kenya: a case-control study. Tropical Med Int Health.

[24] Nanji AA, Mercado C, Galor A, Dubovy S, Karp CL (2017). Updates in ocular surface tumor diagnostics. Int Ophthalmol Clin.

[25] Sayed-Ahmed IO, Palioura S, Galor A, Karp CL (2017). Diagnosis and medical management of ocular surface squamous neoplasia. Expert Rev Ophthalmol.

[26] Oellers P, Karp CL, Sheth A, Kao AA, Abdelaziz A, Matthews JL (2013). Prevalence, treatment, and outcomes of coexistent ocular surface squamous neoplasia and pterygium. Ophthalmology..

[27] Romero IL, Barros Jde N, Martins MC, Ballalai PL (2013). The use of 1% toluidine blue eye drops in the diagnosis of ocular surface squamous neoplasia. Cornea..

[28] Kaji Y, Hiraoka T, Oshika T (2006). Vital staining of squamous cell carcinoma of the conjunctiva using toluidine blue. Acta Ophthalmol Scand.

[29] Gichuhi S, Macharia E, Kabiru J, Zindamoyen AM, Rono H, Ollando E (2015). Toluidine blue 0.05% vital staining for the diagnosis of ocular surface squamous neoplasia in Kenya. JAMA Ophthalmol..

[30] Iovieno A, Longo C, De Luca M, Piana S, Fontana L, Ragazzi M (2016). Fluorescence confocal microscopy for ex vivo diagnosis of conjunctival tumors: a pilot study. Am J Ophthalmol.

[31] Thomas BJ, Galor A, Nanji AA, El Sayyad F, Wang J, Dubovy SR (2014). Ultra high-resolution anterior segment optical coherence tomography in the diagnosis and management of ocular surface squamous neoplasia. Ocul Surf..

[32] Wang J, Abou Shousha M, Perez VL, Karp CL, Yoo SH, Shen M (2011). Ultra-high resolution optical coherence tomography for imaging the anterior segment of the eye. Ophthalmic Surg Lasers Imaging.

[33] Nanji AA, Sayyad FE, Galor A, Dubovy S, Karp CL (2015). High-resolution optical coherence tomography as an adjunctive tool in the diagnosis of corneal and conjunctival pathology. Ocul Surf..

[34] Kieval JZ, Karp CL, Abou Shousha M, Galor A, Hoffman RA, Dubovy SR (2012). Ultra-high resolution optical coherence tomography for differentiation of ocular surface squamous neoplasia and pterygia. Ophthalmology..

[35] Atallah M, Joag M, Galor A, Amescua G, Nanji A, Wang J (2017). Role of high resolution optical coherence tomography in diagnosing ocular surface squamous neoplasia with coexisting ocular surface diseases. Ocul Surf..

[36] Karp CL (2017). Evolving technologies for lid and ocular surface neoplasias: is optical biopsy a reality?. JAMA Ophthalmol.

[37] Parrozzani R, Lazzarini D, Dario A, Midena E (2011). In vivo confocal microscopy of ocular surface squamous neoplasia. Eye (Lond)..

[38] Ong SS, Vora GK, Gupta PK (2016). Anterior segment imaging in ocular surface squamous neoplasia. J Ophthalmol.

[39] Singh S, Mittal R, Ghosh A, Tripathy D, Rath S (2018). High-resolution anterior segment optical coherence tomography in intraepithelial versus invasive ocular surface squamous neoplasia. Cornea..

[40] Xu Y, Zhou Z, Xu Y, Wang M, Liu F, Qu H (2012). The clinical value of in vivo confocal microscopy for diagnosis of ocular surface squamous neoplasia. Eye (Lond).

[41] Adler E, Turner JR, Stone DU (2013). Ocular surface squamous neoplasia: a survey of changes in the standard of care from 2003 to 2012. Cornea..

[42] Stone DU, Butt AL, Chodosh J (2005). Ocular surface squamous neoplasia: a standard of care survey. Cornea..

[43] Shields JA, Shields CL, De Potter P (1997). Surgical management of conjunctival tumors. The 1994 Lynn B. McMahan lecture. Arch Ophthalmol.

[44] Pe'er J (2015). Ocular surface squamous neoplasia: evidence for topical chemotherapy. Int Ophthalmol Clin.

[45] Sepulveda R, Pe'er J, Midena E, Seregard S, Dua HS, Singh AD (2010). Topical chemotherapy for ocular surface squamous neoplasia: current status. Br J Ophthalmol.

[46] Sarici AM, Arvas S, Pazarli H (2013). Combined excision, cryotherapy, and intraoperative mitomycin C (EXCRIM) for localized intraepithelial and squamous cell carcinoma of the conjunctiva. Graefes Arch Clin Exp Ophthalmol.

[47] Viani GA, Fendi LI (2017). Adjuvant treatment or primary topical monotherapy for ocular surface squamous neoplasia: a systematic review. Arq Bras Oftalmol.

[48] Tabin G, Levin S, Snibson G, Loughnan M, Taylor H (1997). Late recurrences and the necessity for long-term follow-up in corneal and conjunctival intraepithelial neoplasia. Ophthalmology..

[49] Houglum JE (1983). Interferon: mechanisms of action and clinical value. Clin Pharm.

[50] Karp CL, Galor A, Chhabra S, Barnes SD, Alfonso EC (2010). Subconjunctival/perilesional recombinant interferon alpha2b for ocular surface squamous neoplasia: a 10-year review. Ophthalmology..

[51] Shah SU, Kaliki S, Kim HJ, Lally SE, Shields JA, Shields CL (2012). Topical interferon alfa-2b for management of ocular surface squamous neoplasia in 23 cases: outcomes based on American joint committee on Cancer classification. Arch Ophthalmol.

[52] Vann RR, Karp CL (1999). Perilesional and topical interferon alfa-2b for conjunctival and corneal neoplasia. Ophthalmology..

[53] Galor A, Karp CL, Chhabra S, Barnes S, Alfonso EC (2010). Topical interferon alpha 2b eye-drops for treatment of ocular surface squamous neoplasia: a dose comparison study. Br J Ophthalmol.

[54] Shields Carol L., Kaliki Swathi, Kim H. Jane, Al-Dahmash Saad, Shah Sanket U., Lally Sara E., Shields Jerry A. (2013). Interferon for Ocular Surface Squamous Neoplasia in 81 Cases. Cornea.

[55] Aldave AJ, Nguyen A (2007). Ocular surface toxicity associated with topical interferon alpha-2b. Br J Ophthalmol.

[56] Arnaud P (2002). Different interferons: pharmacology, pharmacokinetics, proposed mechanisms, safety and side effects. La Revue de Médecine Interne..

[57] Kusumesh R, Ambastha A, Sinha B, Kumar R (2015). Topical interferon alpha-2b as a single therapy for primary ocular surface squamous neoplasia. Asia Pac J Ophthalmol (Phila).

[58] Nanji AA, Moon CS, Galor A, Sein J, Oellers P, Karp CL (2014). Surgical versus medical treatment of ocular surface squamous neoplasia: a comparison of recurrences and complications. Ophthalmology..

[59] Bracarda S, Eggermont AM, Samuelsson J (2010). Redefining the role of interferon in the treatment of malignant diseases. Eur J Cancer.

[60] Edwards L, Levine N, Smiles KA (1990). The effect of topical interferon alpha 2b on actinic keratoses. J Dermatol Surg Oncol.

[61] Woo ASJ, Kwok R, Ahmed T (2017). Alpha-interferon treatment in hepatitis B. Ann Transl Med.

[62] Jang ES, Kim YS, Kim KA, Lee YJ, Chung WJ, Kim IH (2017). Final report of unmet needs of interferon-based therapy for chronic hepatitis C in Korea: basis for moving into the direct-acting antiviral era. Gut Liver.

[63] Mitsuyasu Ronald T. (1991). Interferon alpha in the treatment of AIDS-related Kaposi's sarcoma. British Journal of Haematology.

[64] Kitano S (1984). Clinical trials of interferon in ophthalmology. Gan To Kagaku Ryoho.

[65] Yousef YA, Finger PT (2012). Squamous carcinoma and dysplasia of the conjunctiva and cornea: an analysis of 101 cases. Ophthalmology..

[66] Siedlecki AN, Tapp S, Tosteson AN, Larson RJ, Karp CL, Lietman T (2016). Surgery versus interferon alpha-2b treatment strategies for ocular surface squamous neoplasia: a literature-based decision analysis. Cornea..

[67] Schechter BA, Schrier A, Nagler RS, Smith EF, Velasquez GE (2002). Regression of presumed primary conjunctival and corneal intraepithelial neoplasia with topical interferon alpha-2b. Cornea..

[68] Longley DB, Harkin DP, Johnston PG (2003). 5-fluorouracil: mechanisms of action and clinical strategies. Nat Rev Cancer.

[69] Joag MG, Sise A, Murillo JC, Sayed-Ahmed IO, Wong JR, Mercado C (2016). Topical 5-fluorouracil 1% as primary treatment for ocular surface squamous neoplasia. Ophthalmology..

[70] Parrozzani R, Lazzarini D, Alemany-Rubio E, Urban F, Midena E (2011). Topical 1% 5-fluorouracil in ocular surface squamous neoplasia: a long-term safety study. Br J Ophthalmol.

[71] Venkateswaran N, Mercado C, Galor A, Karp CL (2019). Comparison of topical 5-fluorouracil and interferon alfa-2b as primary treatment modalities for ocular surface squamous neoplasia. Am J Ophthalmol.

[72] Yeatts PR, Engelbrecht NE, Curry CD, Ford JG, Walter KA (2000). 5-fluorouracil for the treatment of tntraepithelial neoplasia of the conjunctiva and cornea. Ophthalmology..

[73] Midena E. (2000). Treatment of conjunctival squamous cell carcinoma with topical 5-fluorouracil. British Journal of Ophthalmology.

[74] Chaugule SS, Park J, Finger PT (2018). Topical chemotherapy for giant ocular surface squamous neoplasia of the conjunctiva and cornea: is surgery necessary?. Indian J Ophthalmol.

[75] Werschler WP (2008). Considerations for use of fluorouracil cream 0.5% for the treatment of actinic keratosis in elderly patients. J Clin Aesthet Dermatol.

[76] Panczyk M (2014). Pharmacogenetics research on chemotherapy resistance in colorectal cancer over the last 20 years. World J Gastroenterol.

[77] Takahashi H, Sakakura K, Mito I, Ida S, Chikamatsu K (2016). Dynamic changes in immune cell profile in head and neck squamous cell carcinoma: immunomodulatory effects of chemotherapy. Cancer Sci.

[78] Abraham LM, Selva D, Casson R, Leibovitch I (2007). The clinical applications of fluorouracil in ophthalmic practice. Drugs..

[79] Verweij J, Pinedo HM (1990). Mitomycin C: mechanism of action, usefulness and limitations. Anti-Cancer Drugs.

[80] Singh P (2013). Mitomycin-C use in ophthalmology. IOSR J Pharmacy.

[81] Rudkin AK, Dempster L, Muecke JS (2015). Management of diffuse ocular surface squamous neoplasia: efficacy and complications of topical chemotherapy. Clin Exp Ophthalmol.

[82] Blasi MA, Maceroni M, Sammarco MG, Pagliara MM (2018). Mitomycin C or interferon as adjuvant therapy to surgery for ocular surface squamous neoplasia: comparative study. Eur J Ophthalmol.

[83] Shields CL, Naseripour M, Shields JA (2002). Topical mitomycin C for extensive, recurrent conjunctival-corneal squamous cell carcinoma. Am J Ophthalmol.

[84] Giacconi JA, Karp CL (2003). Current treatment options for conjunctival and corneal intraepithelial neoplasia. Ocul Surf.

[85] Frucht-Pery J, Sugar J, Baum J, Sutphin JE, Pe’er J, Savir H (1997). Mitomycin C treatment for conjunctival-corneal intraepithelial neoplasia : a multicenter experience. Ophthalmology..

[86] Lee JH, Kim YH, Kim MS, Kim EC (2014). The effect of surgical wide excision and amniotic membrane transplantation with adjuvant topical mitomycin C treatment in recurrent conjunctival--corneal intraepithelial neoplasia. Semin Ophthalmol.

[87] Hirst LW (2007). Randomized controlled trial of topical mitomycin C for ocular surface squamous neoplasia: early resolution. Ophthalmology..

[88] Prabhasawat P, Tarinvorakup P, Tesavibul N, Uiprasertkul M, Kosrirukvongs P, Booranapong W (2005). Topical 0.002% mitomycin C for the treatment of conjunctival corneal intraepithelial neoplasia and squamous cell carcinoma. Cornea..

[89] Ballalai PL, Erwenne CM, Martins MC, Lowen MS, Barros JN (2009). Long-term results of topical mitomycin C 0.02% for primary and recurrent conjunctival-corneal intraepithelial neoplasia. Ophthalmic Plast Reconstr Surg.

[90] Siganos CS, Kozobolis VP, Christodoulakis EV (2002). The intraoperative use of mitomycin-C in excision of ocular surface neoplasia with or without limbal autograft transplantation. Cornea..

[91] Kemp EG, Harnett AN, Chatterjee S (2002). Preoperative topical and intraoperative local mitomycin C adjuvant therapy in the management of ocular surface neoplasias. Br J Ophthalmol.

[92] Chen C, Louis D, Dodd T, Muecke J (2004). Mitomycin C as an adjunct in the treatment of localised ocular surface squamous neoplasia. Br J Ophthalmol.

[93] Quebbeman EJ, Hoffman NE, Ausman RK, Hamid AA (1985). Stability of mitomycin admixtures. Am J Hosp Pharm.

[94] Khong JJ, Muecke J (2006). Complications of mitomycin C therapy in 100 eyes with ocular surface neoplasia. Br J Ophthalmol.

[95] Billing K, Karagiannis A, Selva D (2003). Punctal-canalicular stenosis associated with mitomycin-C for corneal epithelial dysplasia. Am J Ophthalmol.

[96] Daniell M, Maini R, Tole D (2002). Use of mitomycin C in the treatment of corneal conjunctival intraepithelial neoplasia. Clin Exp Ophthalmol.

[97] Wilson MW, Hungerford JL, George SM, Madreperla SA (1997). Topical mitomycin C for the treatment of conjunctival and corneal epithelial dysplasia and neoplasia. Am J Ophthalmol.

[98] Kusumesh R, Ambastha A, Kumar S, Sinha BP, Imam N (2017). Retrospective comparative study of topical interferon alpha2b versus mitomycin c for primary ocular surface squamous neoplasia. Cornea..

[99] Maffezzini M, Campodonico F, Canepa G, Manuputty EE, Tamagno S, Puntoni M (2014). Intravesical mitomycin C combined with local microwave hyperthermia in non-muscle-invasive bladder cancer with increased European Organization for Research and Treatment of Cancer (EORTC) score risk of recurrence and progression. Cancer Chemother Pharmacol.

[100] Tahover E, Bar-Shalom R, Sapir E, Pfeffer R, Nemirovsky I, Turner Y (2018). Chemo-radiotherapy of oligometastases of colorectal cancer with pegylated liposomal mitomycin-c prodrug (promitil): mechanistic basis and preliminary clinical experience. Front Oncol.

[101] Schunke KJ, Rosati LM, Zahurak M, Herman JM, Narang AK, Usach I (2017). Long-term analysis of 2 prospective studies that incorporate mitomycin C into an adjuvant chemoradiation regimen for pancreatic and periampullary cancers. Adv Radiat Oncol.

[102] Miranda MB, Hartmann JT, Al-Batran SE, Kripp M, Gencer D, Hochhaus A (2014). Mitomycin C and capecitabine in pretreated patients with metastatic gastric cancer: a multicenter phase II study. J Cancer Res Clin Oncol.

[103] Klaassen I, Braakhuis BJ (2002). Anticancer activity and mechanism of action of retinoids in oral and pharyngeal cancer. Oral Oncol.

[104] Herbort CP, Zografos L, Zwingli M, Schoeneieh M (1988). Topical retinoic acid in dysplastic and metaplastic keratinization of corneoconjunctival epithelium. Graefes Arch Clin Exp Ophthalmol.

[105] Krilis M, Tsang H, Coroneo M (2012). Treatment of conjunctival and corneal epithelial neoplasia with retinoic acid and topical interferon alfa-2b: long-term follow-up. Ophthalmology..

[106] Nanji AA, Sayyad FE, Karp CL (2013). Topical chemotherapy for ocular surface squamous neoplasia. Curr Opin Ophthalmol.

[107] Skippen B, Tsang HH, Assaad NN, Coroneo MT (2010). Rapid response of refractory ocular surface dysplasia to combination treatment with topical all-trans retinoic acid and interferon alfa-2b. Arch Ophthalmol.

[108] Orfanos CE, Zouboulis CC, Almond-Roesler B, Geilen CC (1997). Current use and future potential role of retinoids in dermatology. Drugs..

[109] Muindi JR, Frankel SR, Huselton C, DeGrazia F, Garland WA, Young CW (1992). Clinical pharmacology of oral all-trans retinoic acid in patients with acute promyelocytic leukemia. Cancer Res.

[110] Sommer A (1983). Effects of vitamin a deficiency on the ocular surface. Ophthalmology..

[111] Harlev E, Nevo E, Lansky EP, Ofir R, Bishayee A (2012). Anticancer potential of aloes: antioxidant, antiproliferative, and immunostimulatory attributes. Planta Med.

[112] Damani MR, Shah AR, Karp CL, Orlin SE (2015). Treatment of ocular surface squamous neoplasia with topical Aloe vera drops. Cornea..

[113] Davis RH, Donato JJ, Hartman GM, Haas RC (1994). Anti-inflammatory and wound healing activity of a growth substance in Aloe vera. J Am Podiatr Med Assoc.

[114] Zabawski EJ, Cockerell CJ (1998). Topical and intralesional cidofovir: a review of pharmacology and therapeutic effects. J Am Acad Dermatol.

[115] Sherman MD, Feldman KA, Farahmand SM, Margolis TP (2002). Treatment of conjunctival squamous cell carcinoma with topical cidofovir. Am J Ophthalmol.

[116] Toro JR, Sanchez S, Turiansky G, Blauvelt A (2003). Topical cidofovir for the treatment of dermatologic conditions: verruca, condyloma, intraepithelial neoplasia, herpes simplex and its potential use in smallpox. Dermatol Clin.

[117] Vossen MG, Gattringer KB, Jäger W, Kraff S, Thalhammer F (2014). Single-dose pharmacokinetics of cidofovir in continuous venovenous hemofiltration. Antimicrob Agents Chemother.

[118] Rahhal FM, Arevalo JF, Munguia D, Taskintuna I, Chavez de la Paz E, Azen SP (1996). Intravitreal cidofovir for the maintenance treatment of cytomegalovirus retinitis. Ophthalmology..

[119] Kim LA, D'Amore PA (2012). A brief history of anti-VEGF for the treatment of ocular angiogenesis. Am J Pathol.

[120] Asena Leyla, Dursun Altınörs Dilek (2015). Topical Bevacizumab for the Treatment of Ocular Surface Squamous Neoplasia. Journal of Ocular Pharmacology and Therapeutics.

[121] Faramarzi A, Feizi S (2013). Subconjunctival bevacizumab injection for ocular surface squamous neoplasia. Cornea..

[122] Paul S, Stone DU (2012). Intralesional bevacizumab use for invasive ocular surface squamous neoplasia. J Ocul Pharmacol Ther.

[123] Finger PT, Chin KJ (2012). Refractory squamous cell carcinoma of the conjunctiva treated with subconjunctival ranibizumab (Lucentis): a two-year study. Ophthalmic Plast Reconstr Surg.

[124] Koenig Y, Bock F, Horn F, Kruse F, Straub K, Cursiefen C (2009). Short- and long-term safety profile and efficacy of topical bevacizumab (Avastin) eye drops against corneal neovascularization. Graefes Arch Clin Exp Ophthalmol.

[125] Hurwitz H, Saini S (2006). Bevacizumab in the treatment of metastatic colorectal cancer: safety profile and management of adverse events. Semin Oncol.

[126] Rosenfeld PJ, Brown DM, Heier JS, Boyer DS, Kaiser PK, Chung CY (2006). Ranibizumab for neovascular age-related macular degeneration. N Engl J Med.

[127] Sinawat S, Rattanapakorn T, Sanguansak T, Yospaiboon Y, Sinawat S (2013). Intravitreal bevacizumab for proliferative diabetic retinopathy with new dense vitreous hemorrhage after full panretinal photocoagulation. Eye (Lond)..

[128] Goyal S, Lavalley M, Subramanian ML (2011). Meta-analysis and review on the effect of bevacizumab in diabetic macular edema. Graefes Arch Clin Exp Ophthalmol.

[129] Ashkenazy N, Karp CL, Wang G, Acosta CM, Galor A (2017). Immunosuppression as a possible risk factor for interferon nonresponse in ocular surface squamous neoplasia. Cornea..

